# Association Between Weekly Moderate-to-Vigorous Physical Activity and Emotional Intelligence Factors in Spanish Adolescents: Perspectives for Digital and Gamified Interventions

**DOI:** 10.3390/jintelligence14010005

**Published:** 2026-01-03

**Authors:** Alberto Ruiz-Ariza, José Enrique Moral-García, Alba Rusillo-Magdaleno, Jose Luis Solas-Martínez

**Affiliations:** 1 Department of Didactic of Musical, Plastic, and Corporal Expression, University of Jaén, 23071 Jaén, Spain; arariza@ujaen.es (A.R.-A.); jsolas@ujaen.es (J.L.S.-M.); 2 Institute for Transfer and Research (ITEI), International University of La Rioja (UNIR), 26006 Logroño, Spain; alba.rusillomagdaleno@unir.net

**Keywords:** emotional competence, physical activity behavior, psychological health, secondary education, self-regulation, digital interventions, wearable technology

## Abstract

This study aimed to analyze the relationship between moderate to vigorous physical activity (MVPA) and dimensions of emotional intelligence (EI) in Spanish adolescents aged 12 to 16 years, controlling for variables such as gender, age, and body mass index (BMI). A total of 171 students (92 boys; mean age = 13.73 ± 1.34 years) were analyzed, measuring MVPA using the PACE + Adolescent PA Measure and EI using the TEIQue-SF, which includes well-being, self-control, emotionality, and sociability. Physically active adolescents (>4 days/week with ≥60 min of MVPA) showed significantly higher scores in well-being and sociability compared to their inactive peers (*p* < 0.05), with no differences in emotionality or self-control. Regression analyses confirmed that weekly MVPA was positively associated with well-being and sociability, independent of gender, age, and BMI. These findings suggest that regular MVPA is associated with emotional balance and social competence in adolescents, highlighting the importance of integrating structured PA programs in and out of school. Furthermore, the study underscores the potential of digital and gamified interventions, such as exergames and mobile apps, as promising tools to support the emotional and social correlates of PA by promoting motivation, social interaction, and emotional regulation, offering innovative approaches to support adolescents’ social-emotional development.

## 1. Introduction

In Spain, the prevalence of sedentary behavior among adolescents continues to represent a major public health concern (Ramírez-Espejo et al., 2025). The most recent national reports consistently indicate that a large proportion of Spanish youth fail to meet the minimum levels of physical activity (PA) recommended by the World Health Organization (WHO). National studies reveal that among adolescents aged 13 to 15 years, sedentary time increases with age, whereas light and moderate PA decline progressively (Fernández-Argüelles et al., 2025). PA is defined as any bodily movement produced by skeletal muscles that requires energy expenditure, encompassing school-based, leisure, and transport-related activities (D’Agostino & Neshteruk, 2023). During adolescence, particularly between 12 and 16 years of age, PA plays a key role in physical, cognitive, and emotional maturation, and is recognized as a reliable predictor of long-term health outcomes (Huang et al., 2023). The WHO recommends that adolescents engage in at least 60 min of moderate-to-vigorous physical activity (MVPA) daily, combining aerobic exercise with muscle- and bone-strengthening activities at least three times per week (Romero-Parra et al., 2023; World Health Organization, 2020). However, adherence to these recommendations depends on multiple individual and contextual factors, including gender, motivation, socioeconomic background, and the surrounding social environment, which together shape adolescents’ PA patterns (Benítez-Sillero et al., 2025). In addition, the pervasive presence of digital technologies in adolescents’ daily lives, ranging from recreational screen use to fitness tracking, has reshaped PA patterns and lifestyle behaviors in this population (Aubert et al., 2022).

According to recent analyses from the international Global Matrix initiative, only between 20% and 39% of Spanish adolescents achieve the minimum recommended 60 min of daily MVPA (Aubert et al., 2022; López-Gil et al., 2023). This means that approximately six to eight out of every ten adolescents in Spain fail to meet the WHO guidelines, while average screen time reaches between 4 and 6 h per day (more than double the recommended maximum of two hours) and overall sedentary behavior frequently exceeds eight hours daily. This highlights the paradox of a generation highly engaged with digital media but insufficiently active from a physical perspective, suggesting the need for innovative technology-based strategies that transform screen time into active, health-promoting experiences (Marques et al., 2023; Pérez-Jorge et al., 2024). Similar regional trends have been identified across the country. For instance, in the Region of Murcia, barely one in three adolescents engages in sufficient PA, and nearly two-thirds exceed recommended sedentary time (López-Gil et al., 2024). In Extremadura, the situation is even more alarming: fewer than one in five adolescents achieve the daily 60 min MVPA target, and over 70% spend most of their leisure time in sedentary pursuits (Mendoza-Muñoz et al., 2024).

Given this situation, understanding the comprehensive benefits of PA during adolescence is essential. From a physiological perspective, regular exercise enhances cardiovascular, muscular, and bone health (Chen et al., 2024; Gao et al., 2023; Lapas-Dias et al., 2022), improves body composition (J. Li et al., 2025), and develops motor coordination (Zaragas et al., 2023), all of which are crucial during pubertal growth (Gmmash et al., 2023). Psychologically, evidence links PA to greater subjective well-being (P. Wei et al., 2025), higher self-esteem (X. Wei et al., 2024), lower stress, and reduced depressive symptoms, acting as a strong modulator of emotional balance (Grava de Moraes et al., 2025; Laurier et al., 2024; Ruiz-Ranz & Asín-Izquierdo, 2025). Adolescents who are more physically active also exhibit lower psychological distress and greater self-confidence (Laurier et al., 2024). Cognitively, aerobic and multicomponent PA has been linked to improved executive functioning, attention, and working memory, which in turn favor academic performance and emotional regulation (Ye et al., 2024; Ben Ezzdine et al., 2025; Morales et al., 2024). Moreover, emerging approaches in educational and health contexts increasingly combine physical and digital components, such as gamified challenges or wearable-based feedback systems, to enhance motivation, adherence, and emotional engagement during PA (Gkintoni et al., 2024; Willinger et al., 2024).

Among all PA intensities, MVPA consistently yields the greatest physical, cognitive, and emotional benefits in adolescents. Evidence indicates that weekly engagement in MVPA, rather than sporadic activity, is associated with better emotional balance, higher well-being, and enhanced executive performance (Ludyga et al., 2022). In educational settings, regular MVPA, both within Physical Education lessons and extracurricular programs, improves cardiorespiratory fitness and cognitive processing speed, particularly when incorporating high-intensity interval training (Pérez-Ramírez et al., 2025). Furthermore, this intensity level has been linked to better health-related quality of life and lower perceived stress, partly mediated by emotional intelligence (EI) (Q. Li et al., 2024).

Within the school context, PA is not only a vehicle for physical and cognitive growth but also a medium for socio-emotional learning. Beyond its physiological and cognitive impact, MVPA also exerts an important psychological influence, particularly on emotional regulation and interpersonal functioning (Extremera et al., 2023; Jackson et al., 2024). Participation in structured and cooperative PA enhances empathy, self-awareness, and emotional self-regulation, which are closely tied to EI (Jackson et al., 2024). This association is underpinned by the capacity of physical exercise to activate neuropsychological processes related to impulse control, stress regulation, and self-perception (Wu et al., 2022; Skurvydas et al., 2021). EI, defined as the ability to perceive, understand, use, and regulate one’s own emotions and those of others (Reyes-Wapano, 2021), plays a critical role during adolescence, a period marked by rapid identity formation and emotional fluctuation (Chamizo-Nieto et al., 2021). Adequate emotional regulation fosters resilience, psychological well-being, and mental health protection, serving as a preventive buffer against stress and depressive symptoms (Extremera et al., 2023). Taken together, these findings highlight the need to explore how PA contributes not only to physical and cognitive outcomes but also to adolescents’ emotional development (Extremera et al., 2023; Jackson et al., 2024).

The interaction between PA and EI has therefore emerged as a key topic in educational and health research, as growing evidence supports a positive association between both constructs, reflecting the intersection of physical well-being and socio-emotional development (Ruiz-Ariza et al., 2019). Studies have shown that regular MVPA enhances EI, fostering emotional regulation, empathy, and stress management (Castro-Sánchez et al., 2022). More recent evidence confirms that participation in structured PA programs improves EI and subjective well-being, reducing stress and strengthening interpersonal relationships (Yang et al., 2024). In this regard, PA can be viewed as a psychological modulator that enhances emotional clarity, attention, and repair, promoting affective stability and positive school relationships (Portela-Pino et al., 2022; San Román-Mata et al., 2020). Moreover, EI has been shown to mediate the relationship between PA and health-related quality of life, suggesting that more active adolescents regulate emotions more effectively and experience lower stress levels (Q. Li et al., 2024). However, few studies have jointly analyzed the differences among active-inactive adolescents and EI levels, according to days with 60 min at moderate to vigorous intensity, and the relationship between weekly MVPA intensity and EI domains in Spanish adolescents, while controlling for critical covariates such as gender, age, and body mass index (BMI). This gap is especially relevant because understanding how weekly MVPA is associated with some emotional components, such as well-being or sociability, may provide new insights into promoting mental health and socio-emotional development during adolescence.

The comparison between physically active and inactive adolescents is particularly relevant due to growing evidence that PA levels influence not only physical health but also the cognitive, emotional, and social development of adolescents. Previous research has shown that PA can improve aspects of psychological well-being and emotional regulation (Gabour et al., 2024; Q. Li et al., 2024), although few studies have analyzed the differences between physically active and inactive students with specific IE factors. Differentiating between physically active and inactive adolescents allows for a better understanding of how the amount of PA is differently associated with EI dimensions (Gkintoni et al., 2024; Yang et al., 2024). Studies such as that by Ruiz-Ariza et al. (2019) have pointed out that regular participation in PA not only improves physical health but can also be an important modulator of EI, affecting variables such as empathy and emotional regulation. However, the lack of distinction between levels of PA in many studies limits the ability to identify how differences in the amount of activity may be associated with specific aspects of EI. The present study seeks to fill this gap by explicitly comparing adolescents who meet MVPA recommendations with those who do not, controlling for relevant variables such as gender, age, and BMI.

In this context, the growing digitalization of youth lifestyles has prompted researchers to explore how the integration of digital technologies and gamified PA programs can replicate or even enhance the emotional and social benefits traditionally associated with exercise. However, exergames have proven to be effective tools for stimulating PA and improving psychological well-being among youth (Marques et al., 2023). Systematic reviews indicate that such interventions significantly enhance positive emotions, intrinsic motivation, and emotional regulation, leading to increased happiness, self-esteem, and self-efficacy in adolescents (Gkintoni et al., 2024). Likewise, gamified PA programs and mobile apps that include social interaction and feedback elements have been shown to improve mental health, motivation, and social connection in school contexts (Pérez-Jorge et al., 2024). Innovative interventions such as the KIJANI mobile application, which combines augmented reality with activity tracking, have demonstrated high engagement and perceived benefits for adolescents, enhancing both enjoyment and adherence to active lifestyles (Willinger et al., 2024). Furthermore, school-based exergaming interventions have produced positive psychological effects related to enjoyment, motivation, and attitudes toward physical exercise, suggesting their potential to foster emotional and social competencies through movement-based learning (Quintas & Bustamante, 2023).

Therefore, the aim of this study was to examine the differences in EI between physically active and inactive adolescents, as well as to analyze the association between weekly MVPA and EI factors in adolescents aged 12–16 years, independently of sex, age, and BMI. Additionally, the study aims to discuss how emerging digital and gamified PA environments could be leveraged to promote EI and well-being in youth, providing insights that may guide the design of future technology-based interventions in educational and health contexts.

## 2. Materials and Methods

### 2.1. Participants

A total of 171 Spanish adolescents (92 boys, 53.80%; 79 girls, 46.2%) from four secondary schools in southern Spain participated in this cross-sectional quantitative study. The selection of educational centers was based on convenience, dependent on the institutions’ agreement to participate. Within each center, participants were selected using cluster random sampling of intact classroom groups.

Eligibility was determined based on specific criteria. The inclusion criteria were: (1) being enrolled in the selected secondary schools; (2) being aged between 12 and 16 years; and (3) providing written informed consent from parents or legal guardians. The exclusion criteria included: (1) having physical pathologies or medical contraindications preventing PA practice; (2) being diagnosed with learning disabilities (e.g., ADHD) or developmental/psychological disorders (e.g., autism spectrum or emotional/behavioral disorders) to avoid potential confounding effects on EI development; (3) current use of medication affecting the central nervous system or metabolic rate; and (4) failing to complete at least 90% of the questionnaire items.

The students’ mean age was 13.73 ± 1.34 years, and their mean BMI was 21.34 ± 3.62 kg/m^2^, with an average of 2.81 ± 1.58 days per week of MVPA. Regarding activity levels, 145 participants (85.4%) were classified as physically inactive and 26 participants (14.6%) as physically active. This disproportionate distribution is consistent with national evidence indicating that most adolescents do not meet PA recommendations (Aubert et al., 2022). The MVPA cut-off used to classify participants as physically active (>4 days/week with ≥60 min) was based on previously published methodological criteria (Kongsvold et al., 2025) and was applied to ensure a conservative and conceptually robust classification. Importantly, the use of this stringent classification threshold naturally results in a smaller active group yet increases conceptual validity by clearly differentiating adolescents with sustained MVPA engagement from those with sporadic or insufficient activity, consistent with the prevalence rates reported in the national context (Aubert et al., 2022). Anthropometric and sociodemographic characteristics are detailed in Table 1.

### 2.2. Measures

#### 2.2.1. Emotional Intelligence

EI was evaluated using the Trait Emotional Intelligence Questionnaire–Short Form (TEIQue-SF), developed by Petrides (2009). This self-report inventory has demonstrated robust internal consistency across diverse populations, as confirmed by a recent reliability generalization meta-analysis (α = 0.86; subscales: Well-being = 0.79, Emotionality = 0.70, Sociability = 0.69, Self-control = 0.68; Orhan, 2024). The TEIQue-SF comprises 30 items rated on a 7-point Likert scale ranging from 1 (Completely disagree) to 7 (Completely agree), and evaluates four key dimensions of trait EI: (1) Well-being, which reflects general life satisfaction and optimism (e.g., “I generally don’t find life enjoyable”); (2) Self-control, which assesses emotional regulation and stress management (e.g., “I usually find it difficult to regulate my emotions”); (3) Emotionality, which captures the ability to perceive, express, and understand emotions (e.g., “Expressing my emotions with words is not a problem for me”); and (4) Sociability, which measures interpersonal competence and social influence (e.g., “I can deal effectively with people”). Items 3, 14, 18, and 29 contribute exclusively to the computation of the global trait EI score, which was not considered in the present analysis.

#### 2.2.2. Weekly Practice of Moderate to Vigorous Physical Activity

Baseline levels of PA were evaluated using the PACE + Adolescent Physical Activity Measure developed by Prochaska et al. (2001). This brief self-report instrument includes two items that assess the number of days on which adolescents accumulated at least 60 min of MVPA, one referring to a typical week and the other to the week immediately preceding data collection. The mean value of both responses was calculated to derive an overall weekly MVPA score. The classification of participants followed the recommendations proposed by Kongsvold et al. (2025), defining adolescents as physically active (>4 days per week with ≥60 min of MVPA) and physically inactive (≤4 days per week). In the present sample, 145 participants (85.4%) were categorized as physically inactive and 26 (14.6%) as physically active. The internal consistency of the MVPA questionnaire has been previously supported in studies with Spanish adolescent populations, showing a Cronbach’s α of 0.78 (Martínez-López et al., 2015), which indicates adequate reliability for this measure.

#### 2.2.3. Confounders

Sex, age, and BMI were included as control variables. Information on sex and age was obtained from the official school records provided by the participating educational institutions. BMI was calculated as weight in kilograms divided by height in meters squared (kg/m^2^). Body weight was assessed using a calibrated ASIMED^®^ B-type Class III scale (Barcelona, Spain), and height was measured with a portable SECA 214 stadiometer (SECA^®^ GmbH, Hamburg, Germany). All anthropometric assessments were conducted with participants barefoot and wearing light clothing to ensure precise measurements.

### 2.3. Procedure

To conduct this study, the principals and teaching staff of the participating schools were first informed about the study objectives and procedures. Written informed consent was obtained from the parents or legal guardians of all participants, and both students and their families received a detailed verbal and written explanation of the study’s purpose, procedures, and voluntary nature. To ensure anonymity and confidentiality, each participant was assigned a unique identification code, and all data were handled in accordance with current data protection standards. Data collection took place during regular school hours, within sessions specifically allocated for this research. During these sessions, anthropometric measurements were obtained by trained researchers, followed by the administration of the self-report questionnaires in a structured and supervised format to ensure full comprehension of the items. The study received approval from the Bioethics Committee of the University of Jaén (Code NOV.22/2.PRY.) and was conducted in accordance with Spanish regulations for clinical research involving humans (Law 14/2007, 3 July, on Biomedical Research), the regulations for personal data protection (Organic Law 15/1999), and the ethical principles outlined in the Declaration of Helsinki (2013 version, Fortaleza, Brazil).

### 2.4. Statistical Analysis

Prior to the main analysis, the dataset was screened for missing values and outliers. Missing data were managed through listwise deletion for participants with more than 10% missing responses to ensure data integrity; for those with minor missing data (<5%), the series mean method was applied to preserve sample size without biasing the results. Extreme values were identified using boxplots and the Mahalanobis distance method (*p* < 0.001) to ensure multivariate normality. No significant outliers requiring removal were detected. The comparison of variables between boys and girls was conducted using independent samples *t*-tests for the descriptive analysis of participants. The normality of the data was assessed using the Kolmogorov–Smirnov test. Although statistical tests indicated a deviation from normality (*p* < 0.05), an inspection of skewness and kurtosis values revealed they were within the acceptable range of ±1.5. Homoscedasticity was verified using Levene’s test, which confirmed the equality of variances across groups (*p* > 0.05).

To examine the differences between adolescents who were physically active (>4 days of ≥60 min of MVPA per week) and those who were physically inactive (≤4 days of ≥60 min of MVPA per week) regarding the four dimensions of EI, analyses of covariance (ANCOVA) were performed. Each EI dimension (well-being, self-control, emotionality, and sociability) was used as a dependent variable, and weekly MVPA was entered as a fixed factor. Weekly MVPA was dichotomized as follows: participants who reported more than four days per week with at least 60 min of MVPA (questionnaire score > 4) were classified as physically active, whereas those who reported four or fewer days (questionnaire score ≤ 4) were classified as physically inactive. Given that the comparison groups had different sample sizes, the effect size was calculated using Hedges’ ĝ, interpreted as 0.2 = small effect, 0.5 = medium effect, and 0.8 = large effect (Martínez-López et al., 2018). The percentage difference between groups was calculated as: [(higher measurement − lower measurement)/lower measurement] × 100. Additionally, a multiple linear regression analysis was performed using the enter method, with weekly MVPA as the independent variable and the four factors of EI (well-being, self-control, emotionality, and sociability) as the dependent variables. Multicollinearity was assessed using variance inflation factors (VIF < 5), and model fit was evaluated using the adjusted coefficient of determination (Adjusted R^2^). Age, sex, and BMI were included as covariates in all analyses. All statistical analyses were performed using SPSS version 25.0 (IBM Corp., Armonk, NY, USA), with statistical significance established at *p* < 0.05.

## 3. Results

Table 1 shows the anthropometric and emotional characteristics of the participants. Adolescents had a mean age of 13.73 ± 1.34 years, a mean weight of 56.87 ± 12.57 kg, a height of 1.62 ± 0.09 m, and a BMI of 21.34 ± 3.62 kg/m^2^. On average, participants performed 2.81 ± 1.58 days per week of MVPA. Boys were significantly older (*p* = 0.012) than girls, although no significant differences were found in BMI (*p* = 0.766). Regarding EI, adolescents showed moderately high levels in all factors, with mean scores of 4.36 ± 0.68 for well-being, 4.15 ± 0.75 for self-control, 3.71 ± 0.72 for emotionality, and 4.20 ± 0.80 for sociability. Boys scored slightly higher than girls in sociability (*p* = 0.047), while no significant sex differences were observed in the remaining dimensions (all *p* > 0.05).

### 3.1. Differences in Emotional Intelligence Dimensions Between Physically Active and Inactive Adolescents

**Figure 1:**
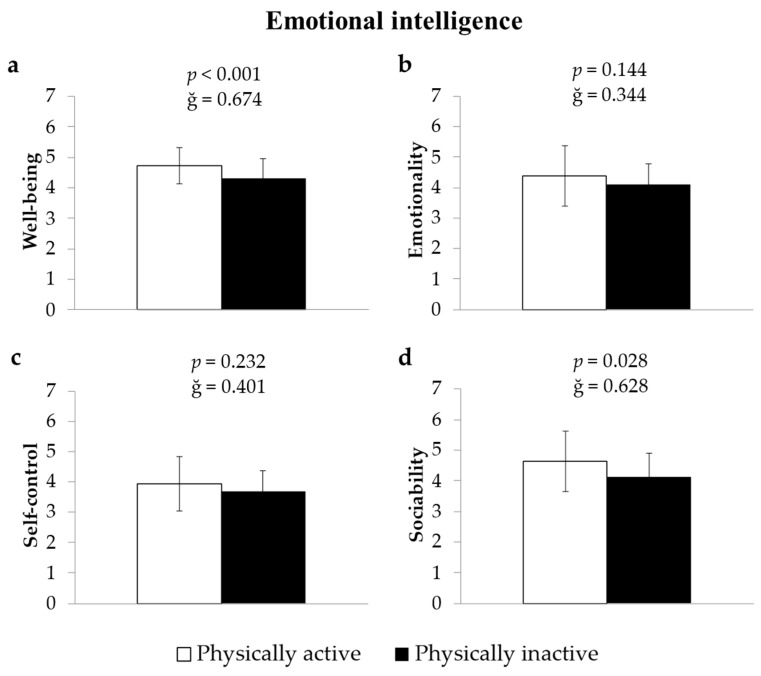
Differences in emotional intelligence dimensions between physically active and inactive adolescents. Note. Values represent means ± standard deviation for each emotional intelligence dimension: (**a**) Well-being, (**b**) Emotionality, (**c**) Self-control, and (**d**) Sociability. Group comparisons were performed using analysis of covariance (ANCOVA), controlling for age, BMI, and sex. The *p*-values indicate the statistical significance of between-group differences, and ĝ denotes the effect size (Hedges’ g).

Adolescents who were physically active (>4 days per week with ≥60 min of MVPA) exhibited significantly higher well-being scores compared to their inactive peers (4.73 ± 0.60 vs. 4.30 ± 0.65 arbitrary units), F(1, 166) = 12.68, *p* < 0.001, ĝ = 0.674, 1 − β = 0.943 (Figure 1a). Likewise, physically active adolescents showed significantly greater sociability (4.63 ± 0.99 vs. 4.12 ± 0.76), F(1, 166) = 4.94, *p* = 0.028, ĝ = 0.628, 1 − β = 0.599 (Figure 1d). No statistically significant differences were observed in emotionality or self-control between active and inactive adolescents (all *p* > 0.05; Figure 1b,c).

### 3.2. Multiple Linear Regression Analysis Between Weekly Moderate-to-Vigorous Physical Activity and Emotional Intelligence Dimensions

Table 2 presents the associations between weekly MVPA and the dimensions of EI, controlling for sex, age, and BMI. Higher weekly MVPA was significantly associated with greater well-being (*B* = 0.075; SE = 0.035; *p* = 0.033) and sociability (*B* = 0.100; SE = 0.042; *p* = 0.018). In addition, higher BMI was significantly associated with greater emotionality (*B* = 0.042; SE = 0.015; *p* = 0.007). None of the remaining associations reached statistical significance (*p* > 0.05).

## 4. Discussion

The aim of this study was to analyze the association between weekly MVPA and the dimensions of EI in adolescents aged 12 to 16 years, controlling for sex, age, and BMI. Although the proportion of physically active adolescents was relatively small, this distribution mirrors real-world patterns of MVPA adherence in adolescence and does not compromise internal validity, as all analyses controlled for key confounders. The results revealed that adolescents who were physically active exhibited significantly higher scores in well-being and sociability compared to their inactive peers. In addition, the regression analyses confirmed that weekly MVPA was positively associated with both well-being and sociability, whereas no significant associations were observed for emotionality or self-control. Overall, these findings indicate that regular engagement in MVPA is related to better emotional functioning, particularly in components related to positive mood and social interaction among adolescents. A key challenge in public health is addressing the significant drop in MVPA adherence in this age group, which is heavily influenced by high screen time and digital engagement (Aubert et al., 2022; López-Gil et al., 2023). Therefore, our findings, which identify Well-being and Sociability as domains positively associated with regular activity, provide the crucial emotional targets for the design of technology-mediated solutions. Specifically, the integration of digital tools and gamification, such as exergames, mobile fitness applications (Willinger et al., 2024), or challenges incorporating rewards and social competition, can leverage established motivational principles (Pérez-Jorge et al., 2024). By focusing on enhancing Well-being through enjoyable challenges and Sociability through team-based or competitive game elements, these gamified approaches offer innovative avenues to support adolescents’ socio-emotional development and promote adherence to active lifestyles (Gkintoni et al., 2024).

These findings are consistent with previous evidence showing that adolescents with higher levels of PA tend to display better emotional adjustment and overall well-being than their less active counterparts. A growing body of literature has demonstrated that regular participation in MVPA is linked to improved health-related quality of life (Alzahrani et al., 2023), greater EI (Gabour et al., 2024; Ruiz-Ariza et al., 2019), lower perceived stress (Q. Li et al., 2024), and enhanced emotional clarity and regulation (Portela-Pino et al., 2022). In line with our results, H. Wang et al. (2023) found that MVPA was positively associated with EI and perceived health status while reducing psychosocial stress in high school students. At the physiological level, these associations may be explained by neuroplastic adaptations resulting from regular MVPA, which stimulate brain regions involved in emotional regulation, such as the orbitofrontal cortex and limbic system (Martínez et al., 2024; Zhang et al., 2023). Moreover, MVPA promotes the release of neurotransmitters and hormones, including oxytocin and endorphins, which facilitate stress reduction and social bonding (Liu et al., 2024; Mennitti et al., 2024). From a psychological perspective, MVPA enhances emotional self-regulation, perceived self-efficacy, and resilience, which in turn mediate the relationship between PA and subjective well-being (Fuentealba-Urra et al., 2023). All these processes improve the ability to identify and manage one’s own and others’ emotions and the processes of emotional regulation and group cohesion (Fuentealba-Urra et al., 2023; Q. Li et al., 2024; Liu et al., 2024; Mennitti et al., 2024; H. Wang et al., 2023).

Nevertheless, despite growing evidence supporting the positive effects of PA on emotional development, some studies have reported inconsistent or weaker relationships. For instance, recent research has suggested that discrepancies may arise from the lack of standardization in intervention programs and limited control over exercise intensity (Francisquini et al., 2024; Ruiz-Ranz & Asín-Izquierdo, 2025; Zhou et al., 2024). Similarly, Jackson et al. (2024) found that improvements in emotional skills were only moderate and not always significant, emphasizing the importance of sustained and integrated approaches over time. Variability across studies may also be attributed to the different tools used to assess EI (León et al., 2024), as self-report measures capture subjective perceptions rather than objective emotional competence (Siregar et al., 2023). Furthermore, cultural and contextual influences may play an important role, given that the social value placed on sport and access to PA opportunities differ between educational and sociocultural environments (Q. Wang et al., 2024; Yang et al., 2024). Finally, from a developmental perspective, some components of EI, such as self-control and emotional regulation, rely heavily on neurocognitive maturation and sustained social experiences (Valenzuela et al., 2025; Deliu & Pérez, 2024), which may not be fully consolidated during early adolescence (Q. Li et al., 2024).

Our findings specifically indicate an association between weekly MVPA and well-being and sociability, but not emotionality or self-control. These differences can be interpreted in light of the distinct mechanisms through which PA is related to emotional processes (Q. Li et al., 2024; Martínez et al., 2024; Yang et al., 2024). While well-being and sociability may benefit directly from the affective and social experiences embedded in PA, emotionality and self-control likely depend on more complex cognitive and self-reflective mechanisms that develop gradually over time. In support of this idea, Gabour et al. (2024) emphasized that higher intensity and longer duration of MVPA are associated with improved self-regulatory abilities, but such effects require consistent practice and structured social–emotional learning contexts. Similarly, H. Wang et al. (2023) demonstrated that emotional self-regulation improved only when exercise programs were integrated with explicit emotional education components. In addition, contextual variables such as family relationships, perceived social support, and school climate have been shown to be more strongly associated with emotionality and self-control than individual levels of PA (Chandran & Nair, 2015).

The associations observed on well-being and sociability are consistent with existing literature and may reflect how PA is related to these more visible aspects of emotional intelligence. Research suggests that physically active adolescents tend to exhibit greater well-being and social skills (Portela-Pino et al., 2022; Gabour et al., 2024). However, although MVPA shows an immediate and visible impact on social and emotional well-being, dimensions such as emotional regulation and self-control require more specific and prolonged interventions, including complementary psychoeducational components (Ludyga et al., 2022; Gabour et al., 2024). In this regard, integrating gamified PA strategies within school or extracurricular programs could help bridge this gap by embedding emotional learning into engaging, interactive, and feedback-rich activities. Such environments may enhance adolescents’ capacity for self-regulation and empathy while maintaining high levels of motivation and adherence (Corepal et al., 2018).

The positive associations observed between weekly MVPA and the EI dimensions of well-being and sociability suggest that digital and gamified PA interventions could be strategically designed to reproduce the affective and social mechanisms underlying these benefits in adolescents (Q. Li et al., 2024; Yang et al., 2024). Systematic reviews indicate that exergames and gamified PA environments enhance enjoyment, positive affect, and intrinsic motivation towards exercise, which are core psychological processes linked to subjective well-being and adherence to active lifestyles in young people (Gkintoni et al., 2024; Marques et al., 2023). School-based exergaming programs have also been shown to improve motivation, attitudes toward PA, and psychological variables related to the promotion of regular exercise, underscoring their potential to support emotionally meaningful movement experiences in educational settings (Quintas & Bustamante, 2023). In parallel, gamified PA interventions delivered through mobile or digital platforms have demonstrated beneficial associations with mental health, emotional functioning, and engagement in adolescents, particularly when they incorporate challenges, rewards, and social interaction components (Pérez-Jorge et al., 2024). Recent multimethod evaluations of applications highlight that combining activity tracking with playful, augmented-reality–based tasks can elicit high levels of enjoyment, perceived usefulness, and sustained participation in children and adolescents, which are key conditions for leveraging PA to foster emotional well-being and social skills (Willinger et al., 2024). Taken together, this evidence suggests that integrating digital and gamified PA tools into school curricula and extracurricular programs, especially those that emphasize cooperative tasks, real-time feedback, and emotionally supportive goal structures, may be a promising pathway to translate the socio-emotional benefits of MVPA into scalable, technology-supported interventions for youth (Gkintoni et al., 2024; Jackson et al., 2024; Pérez-Jorge et al., 2024).

The differences found in well-being and sociability between physically active and inactive adolescents corroborate the idea that the amount of PA is associated with components of EI. In line with previous studies, our findings suggest that regular participation in MVPA is more strongly associated with visible emotional and social aspects of EI, such as well-being and sociability, than with emotionality or self-control. The literature indicates that subjective well-being and sociability benefit primarily from the immediate emotional and social experiences derived from regular PA, particularly in group or cooperative settings (Gabour et al., 2024; Portela-Pino et al., 2022). Furthermore, research such as that by Martínez et al. (2024) has shown that regular PA, particularly MVPA, is associated with the activation of neuropsychological processes related to emotional regulation and self-regulation, which may explain the impact on domains of EI linked to well-being and sociability (Q. Li et al., 2024; Martínez et al., 2024). However, more complex components of EI, such as self-control and emotionality, require more profound neurocognitive maturation, which may not be fully achieved during early adolescence (Q. Li et al., 2024; Martínez et al., 2024).

### Limitations and Strengths

Although this study provides evidence supporting the link between MVPA and specific EI components, several limitations should be acknowledged. First, the cross-sectional design precludes causal inference, so longitudinal and experimental studies are needed to confirm directionality. Second, the sample was selected using a convenience sampling method, which limits the generalizability of the results to the broader adolescent population. Additionally, although the sample reflects the school realities commonly observed in southern Spain, this sampling strategy limits representativeness at the population level, especially with regard to the proportion of adolescents who comply with MVPA recommendations. Third, both MVPA and EI were assessed using self-report questionnaires, which are susceptible to social desirability bias and subjective interpretation. Finally, the present study did not differentiate between types of PA (e.g., individual, team-based, competitive, or recreational), a distinction that may be relevant since collective activities typically foster empathy and social connectedness more effectively than individual exercises (Jackson et al., 2024). Despite these limitations, the study also offers important strengths. It included a school-based sample of Spanish adolescents aged 12–16 years, providing evidence from a Spanish educational setting and offering cultural insights that remain underrepresented in the literature. Additionally, this study specifically examined moderate-to-vigorous activity levels, addressing one of the main methodological gaps identified in prior research, and controlled for key confounding variables such as sex, age, and BMI, thereby strengthening internal validity.

From an applied perspective, the present findings reinforce the importance of promoting regular MVPA within both school curricula and extracurricular contexts as a means to strengthen adolescents’ emotional well-being and social competence. Beyond traditional PA formats, the growing evidence on digital and gamified approaches suggests that hybrid interventions combining structured physical exertion with interactive technologies may amplify these benefits. Such tools—ranging from exergames to mobile applications and virtual activity challenges—can increase motivation, social connectedness, and emotional engagement, thereby supporting EI dimensions such as well-being and sociability, which were positively associated with weekly MVPA in the present study (Gkintoni et al., 2024; Vorlíček et al., 2024). Incorporating these strategies into educational settings may enhance cooperative behaviors, peer relationships, and affective regulation by embedding movement into meaningful, feedback-rich learning experiences. Future research should prioritize longitudinal and experimental designs to examine the sustainability of these gains and to identify which combinations of traditional and technology-supported PA are most effective for promoting robust socio-emotional development during adolescence.

## 5. Conclusions

In conclusion, this study demonstrates that regular participation in MVPA is positively associated with well-being and sociability in adolescents. These findings suggest that PA is related to emotional balance and social connection during a developmental stage characterized by significant psychological and social change. However, the lack of significant associations with emotionality and self-control indicates that the effects of MVPA on EI are not uniform across all dimensions, likely reflecting the distinct cognitive and regulatory mechanisms underlying each emotional domain. Consequently, this study contributes to consolidating the idea that Physical Education provides a privileged context for adolescent socio-emotional development. In line with current evidence, these results also highlight the potential of integrating digital and gamified PA strategies, such as exergames, mobile applications, and virtual activity challenges, to extend the socio-emotional benefits of MVPA by enhancing motivation, social interaction, and engagement in youth. Future research should explore these relationships through longitudinal and intervention-based designs to clarify causal pathways and determine how traditional and technology-supported PA can jointly foster adolescents’ emotional development over time. It would also be relevant to examine whether the use of new technologies and gamification produces stronger effects on EI than traditional Physical Education.

## Figures and Tables

**Table 1:** Anthropometric characteristics, weekly physical activity, and EI dimensions of participants stratified by sex.

	All (*n* = 171)		Boys (*n* = 92)		Girls (*n* =79)		
Variables	Mean	SD	Mean	SD	Mean	SD	*p*
Age (years)	13.73	1.340	13.96	1.32	13.47	1.26	0.012
Weight (kg)	56.87	12.57	59.31	14.55	54.16	9.26	0.005
Height (m)	1.62	0.093	1.66	0.10	1.59	0.06	<0.001
BMI (kg/m^2^)	21.34	3.62	21.26	3.80	21.43	3.43	0.766
Weekly MVPA (days/week)	2.81	1.58	3.23	1.64	2.30	1.35	<0.001
Emotional intelligence							
Well-being	4.36	0.68	4.32	0.77	4.41	0.57	0.379
Self-Control	4.15	0.75	4.24	0.75	4.06	0.75	0.112
Emotionality	3.71	0.72	3.80	0.73	3.63	0.71	0.111
Sociability	4.20	0.80	4.31	0.83	4.08	0.75	0.047

Note. Data are presented as mean and standard deviation. BMI = Body Mass Index; SD = Standard Deviation.

**Table 2:** Multiple linear regression analysis predicting emotional intelligence dimensions from weekly MVPA.

**Variable**	**Well-Being**				**Emotionality**			
	***B***	**SE**	**95% CI**	***p***	***B***	**SE**	**95% CI**	***p***
Sex	−0.177	0.110	−0.395–0.041	0.110	0.226	0.118	−0.007–0.460	0.058
Age (years)	0.035	0.042	−0.047–0.117	0.402	−0.072	0.045	−0.161–0.016	0.108
BMI (kg/m^2^)	−0.001	0.014	−0.030–0.027	0.925	0.042	0.015	0.012–0.073	0.007
Weekly MVPA (days/week)	0.075	0.035	0.006–0.144	0.033	−0.015	0.038	−0.089–0.059	0.690
Adjusted R^2^	0.008				0.036			
	**Self-Control**				**Sociability**			
	***B***	**SE**	**95% CI**	***p***	***B***	**SE**	**95% CI**	***p***
Sex	0.161	0.126	−0.087–0.409	0.201	0.163	0.132	−0.098–0.424	0.219
Age (years)	−0.024	0.047	−0.117–0.070	0.619	−0.061	0.050	−0.160–0.037	0.221
BMI (kg/m^2^)	0.004	0.016	−0.028–0.037	0.792	0.017	0.017	−0.017–0.051	0.331
Weekly MVPA (days/week)	0.008	0.040	−0.070–0.087	0.838	0.100	0.042	0.017–0.183	0.018
Adjusted R^2^	−0.010				0.056			

Weekly MVPA = moderate-to-vigorous physical activity (average number of days per week with ≥60 min); SE = Standard Error; 95% CI = 95.0% confidence interval. All models were adjusted for sex, age, and BMI. Sex was coded as 0 = girl, 1 = boy.

## Data Availability

The datasets underpinning this study’s results are not accessible to the public due to ethical and confidentiality considerations. As this research forms part of a broader project involving various collaborators, safeguarding participant anonymity was essential to secure their participation. In alignment with ethical standards and to preserve the privacy of respondents, data sharing is not permitted.

## Abbreviations

The following abbreviations are used in this manuscript:

MVPA	Moderate-to-vigorous physical activity
PA	Physical Activity
EI	Emotional Intelligence
